# High particulate matter burden by cigarillos: A laser spectrometric analysis of second-hand smoke of common brands with and without filter

**DOI:** 10.1371/journal.pone.0254537

**Published:** 2021-07-09

**Authors:** Markus Braun, Maike Dehm, Doris Klingelhöfer, David A. Groneberg

**Affiliations:** Institute of Occupational Medicine, Social Medicine and Environmental Medicine, Goethe University, Frankfurt am Main, Germany; University of Calfornia San Francisco, UNITED STATES

## Abstract

Although the global tobacco market of cigarillos is substantial, little is known about their particulate matter (PM) emissions. For exposure risk assessment of cigarillos, the PM fractions PM_10_, PM_2.5_, and PM_1_ of eight cigarillo brands (four with filters) and a reference cigarette were measured. For this purpose, second-hand smoke was generated by an automatic smoke pump in a measuring chamber with a volume of 2.88 m³. The mean particle concentrations of the cigarillos ranged from 2783 μg/m³ to 6686 μg/m³ for PM_10_, from 2767 μg/m³ to 6585 μg/m³ for PM_2.5_, and from 2441 to 4680 μg/m³ for PM_1_. Mean concentrations of the reference cigarette for PM_10_, PM_2.5_, and PM_1_ were 4400 μg/m³, 4335 μg/m³, and 3289 μg/m³, respectively. Filter-tipped cigarillos showed between 5% and 38% lower PM_10_ and PM_2.5_ levels, respectively, and between 4% and 30% lower PM_1_ levels. Our findings show generally high PM emissions for all investigated tobacco products. Therefore, the declaration of PM amounts to government authorities should be mandatory for all tobacco products. Policymakers should ensure that corresponding information will be provided in the future.

## Introduction

The global landscape of tobacco consumption is changing, and the use of non-cigarette products is increasing, particularly for young tobacco consumers. That applies not only to e-cigarettes but also to cigar products. For example, between 2000 and 2015, cigarette consumption in the USA decreased from 435,570 million to 267,043 million pieces per year (-38.7%), while cigar consumption increased from 6,161 million to 11,411 million (+85.2%) [1,2]. In the USA, the most popular cigar product are cigarillos [3]. The *National Adult Tobacco Survey*, *United States*, *2012–2013*, reported that 61.8% of cigar smokers aged ≥18 years smoked cigarillos [4]. This survey also states that 21.3% of persons aged ≥18 years were smokers, and 2% were smokers of cigars, cigarillos, or filtered little cigars [5]. That means that about 1.2% of US American persons aged ≥18 years were cigarillo smokers at the time of the survey. In the USA, it is popular among young adults to use wrappers of cigarillos for “blunts” to roll marijuana cigarettes [6]. The data for Europe is something different. The sales of cigarettes between 2012 and 2020 decreased from 1,193,885 million pieces to 931,220 million (-22%). The sales of cigars also decreased in the same period from 21,192 million to 13,864 million (-34.6%). In Germany, the sales of cigarettes and cigars also decreased from 82,405 million in 2012 to 71,940 million in 2020 (-12.7%), respectively 3,926 million to 2,878 million (-26.7%) [7].

Cigarillos, like all cigars, have a wrapper made of tobacco but are smaller than regular cigars and sometimes only slightly bigger than cigarettes [8]. They often have no filter. In 1999, Boffetta et al. [9] ascertained that the lung cancer risk of cigar and cigarillo smokers is comparable to that of cigarette smokers, while most former studies showed a lower risk [10,11]. These previous studies reasoned that this is due to the usually lower level of inhalation when smoking cigars or cigarillos. In contrast, the findings of a conference in June 1998 of the American Cancer Society are unambiguous: smoking of cigar products is as harmful to health as smoking cigarettes, and the second-hand smoke (SHS) from cigar products potentially contributes more to indoor air pollution than SHS from cigarettes [12]. Two studies from 2015 considered the amount of airborne particulate matter (PM) in SHS emitted by “eco” cigarillos with filters (little cigars) of popular cigarette brands. These are cheaper than regular cigarettes of the same brand due to lower taxation in the European Union (EU) [13,14]. The results of the analysis were higher PM burdens by SHS of “eco” cigarillos also due to a longer burning time. So far, little is known about the PM burden in SHS from “real” cigarillos. Therefore, the present study addressed an investigation of PM emissions from “real” cigarillos with and without filters for exposure risk assessment.

In households of smokers, tobacco smoke, especially SHS, is the main source of indoor PM [15]. PM consists of airborne solid and liquid particles of different sizes, origins, and compositions [16]. The US Environmental Protection Agency (EPA) classifies PM in PM_10_ and PM_2.5_. PM_10_ is defined as inhalable particles with a diameter ≤ 10μm and PM_2.5_ as fine inhalable particles with a diameter ≤ 2.5μm [16]. PM_1_ consists of particles ≤ 1μm, accordingly. The size-related PM classification is the most relevant and characterizes the depth of particle penetration into the respiratory tract. Smaller particles can penetrate deeper into the respiratory system affecting more extensive health effects [17].

In 2016, the World Health Organization (WHO) estimated that PM caused 4.2 million premature deaths worldwide [18]. Thus, PM is an essential trigger of non-communicable diseases, a leading cause of death [19]. Many studies showed a relation between PM exposure and an increase in morbidity and mortality [20]. It is well known that PM as part of air pollution has several adverse health effects, mainly on the cardiovascular and respiratory systems. PM can also provoke neurological or psychiatric disorders, such as autism, anxiety, or attention deficit hyperactivity disorder (ADHD). PM is also associated with an increase in breast cancer mortality [21–23]. Maternal PM exposure during pregnancy is hazardous to the health of the infant [24]. Fine particles ≤ 2.5μm (PM_2.5_) may increase the risk for Alzheimer’s disease and related dementias by altering the brain structure potentially due to the neurotoxicity of PM_2.5_ [25].

## Methods and materials

### Tobacco products

PM emissions of eight cigarillo brands, four without and four with filter (Table 1), were measured and compared to the filtered king-size reference cigarette 3R4F from the Kentucky Tobacco Research and Development Center (University of Kentucky, USA) [26]. The filters of the reference cigarette were ventilated, but those of the filtered cigarillos were not. However, all cigarillos had ventilation holes in the region of the tobacco rods. For the measurements, the filters were not blocked. The filter-tipped cigarillos were the cigarillos B, D, F, and H. Cigarillos A and B, C and D, E and F, and G and H were each of the same brand. All cigarillos were machine-made with filler of shredded tobacco and a wrapper of tobacco leaves. Cigarillos C, D, E, and F had an additional binder made of reconstituted tobacco. All cigarillos were bought in Frankfurt, Germany, and were manufactured in the EU. Some characteristics of the investigated tobacco products in terms of sizes and weights are shown in Table 1. Information regarding the ingredients of the cigarillos is available on the webpage of the Federal Ministry of Food and Agriculture of Germany (BMEL) [27]. However, concerning cigarillos, no information is available on the levels of tar, nicotine, and carbon monoxide (CO). The reported amounts of the reference cigarette are as follows: tar 9.4 mg, nicotine: 0.73 mg, CO: 12 mg [26]. For comparison, the reported amounts of the “regular strong” and popular cigarette brands Marlboro Red and Lucky Strike Original Red are as follows: tar 10 mg, nicotine: 0.8 mg, CO: 10 mg [28,29]. For the series of measurements, 20 pieces of each tobacco product were used.

**Table 1:** Characteristics of the investigated tobacco products.

Brand	Total length [mm]	Filter length [mm]	Diameter [mm]	Tobacco weight [mg]
**Reference cigarette 3R4F**	84	27	8	718
**Cigarillo A: Dannemann Moods**	73	n/a	8	1056
**Cigarillo B: Dannemann Moods Filter**	91	17	8	1164
**Cigarillo C: Panter Desert**	74	n/a	8	950
**Cigarillo D: Panter Desert Filter**	78	20	8	720
**Cigarillo E: Clubmaster Mini Red**	66	n/a	7	818
**Cigarillo F: Clubmaster Mini Red Filter**	69	17	7	710
**Cigarillo G: Al Capone**	82	n/a	8	1330
**Cigarillo H: Al Capone Filter**	83	16	8	986

Dimensions and weights are the mean values of five randomized chosen tobacco products each brand. n/a = not available.

### Automatic Environmental Tobacco Smoke Emitter

To generate SHS in a repeatable way, a programmable Automatic Environmental Tobacco Smoke Emitter (AETSE), a smoke pump for medical research by Schimpf-ING Trondheim [30], was applied. The AETSE with its 200 ml glass syringe was located in a sealed measuring chamber with an internal space volume of 2.88 m³. The interior surfaces of the chamber consisted of glass and PVC. The smoke pump was connected via a polyamide tube and two valves with the mouthpiece of the tobacco product, which was placed 38 cm next to the suction point of the measuring device at the same height. The two valves were for regulating the airstream during suction and exhausting of the smoke. The outlet valve was also placed 38 cm beside the suction point near the tobacco product. According to the smoking protocol, the smoke pump moved by a stepper motor took puffs of the tobacco product. After each puff, the mainstream smoke was injected valve-controlled into the chamber 38 cm near the suction point. The tobacco product smoldered between each puff and caused side-stream smoke. The entire setup ensured reproducible generation of SHS by automatically smoking tobacco products without exposing the person who operated the AETSE from the outside of the measuring chamber.

### Smoking protocol

An adapted protocol was applied following the Tobacco Smoke Particles and Indoor Air Quality (ToPIQ) studies [31]. The AETSE was set for a puff volume of 40 ml, a puff flow rate of 13 ml/s, and a puff frequency of two puffs per minute with two initial puffs and eight regular puffs of each single tobacco product. That resulted in a duration of the combustion phase of 4:30 min where the tobacco product was lighted and smoked. Afterward, the tobacco product was extinguished promptly. After the at least 6-min post-combustion phase, an industrial radial fan ventilated the chamber for at least 5 min to purify the indoor air. That ensured measurement data of exact 10 min and a smoke-free measuring cabin for the following measurement. During the combustion and post-combustion phase, the chamber and the air vents were closed to minimize air exchange. Then the next cycle began starting with a blank measurement.

### Laser Aerosol Spectrometer

The used Laser Aerosol Spectrometer (LAS) and Dust Monitor Model 1.109 of Grimm Aerosol Technik GmbH & Co KG (Ainring, Germany) [32] detects airborne particles in a size range from 32 μm down to 0.25 μm every six seconds in real-time. The smoke that was drawn into the device (side-stream smoke from the smoldering tobacco product and the pressed back mainstream smoke from the smoke pump) was diluted 1:10 with compressed air to prevent a blockage of the measuring chamber of the LAS by high particle amounts. The dilution factor was considered accordingly in the data processing. The LAS displayed the measured values according to the US EPA as PM_10_ and PM_2.5_ values [16] as well as PM_1_ values in μg/m³, and according to the European Standard EN 481 categorized as inhalable, thoracic, and respirable [μg/m³] [33].

### Data processing

For the following statistical data processing, including calculation of the mean concentration (C_mean_), data from the 4:30-min lasting combustion phase and 5:30 min lasting post-combustion phase (data of 10 min in sum) were used. After testing for outliers (Grubb’s test) and standard distribution, the data of each tobacco product were tested against each other using a one-way analysis of variance (one-way ANOVA, Kruskal-Wallis test, and Dunn’s multiple comparison test). The level of significance was set at p < 0.05. All statistical tests were performed using GraphPad Prism software (version 8 for Windows, GraphPad Software, La Jolla California USA, www.graphpad.com).

## Results

**Fig 1:**
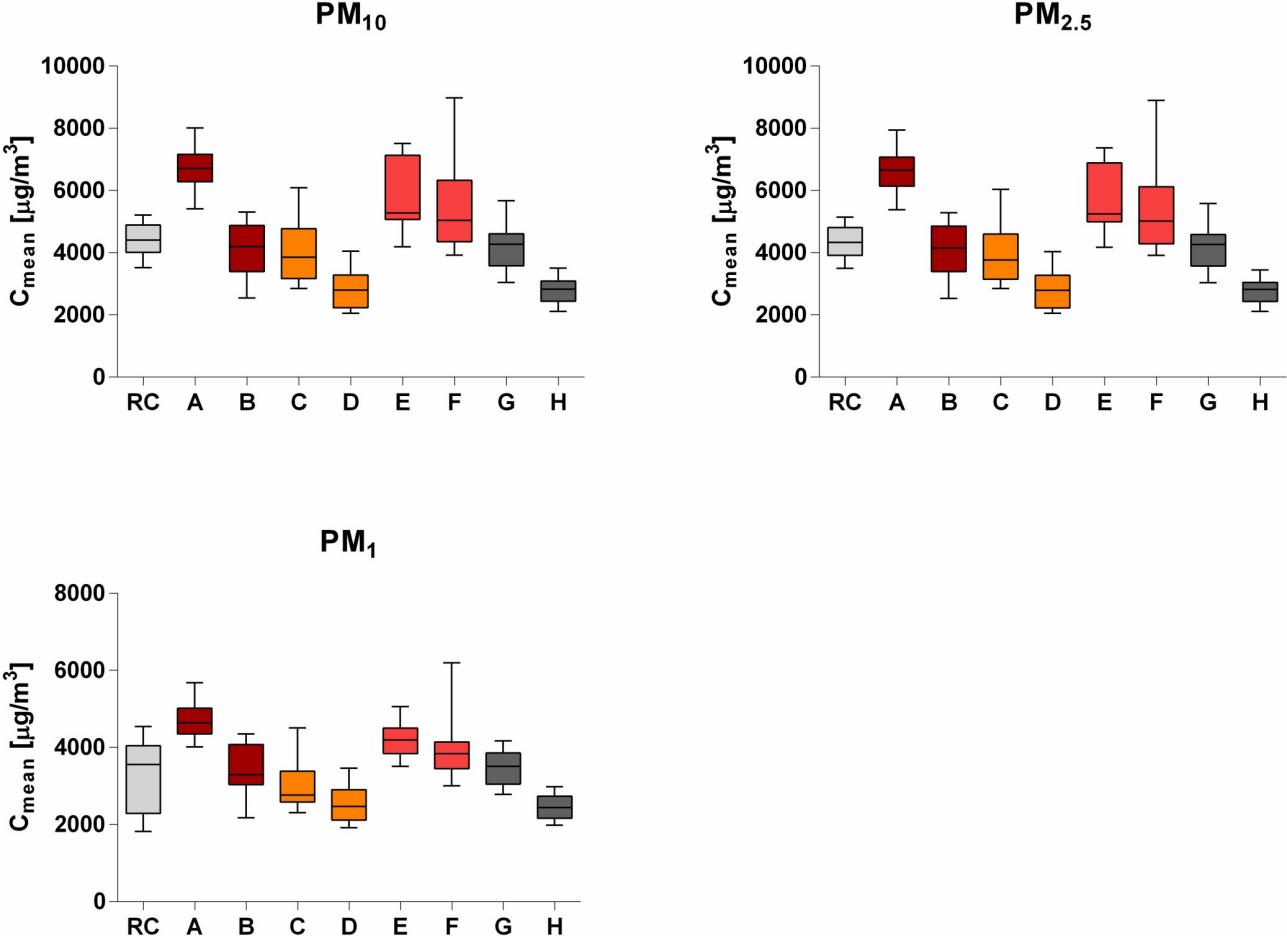
Comparative boxplots (min to max whiskers) of the mean concentrations (C_mean_) of the particle fractions PM_10_, PM_2.5_, and PM_1_ for the reference cigarette 3R4F (RC) and the cigarillos A to H. The cigarillo pairs A and B, C and D, E and F, and G and H are respectively of the same brand. Filter-tipped cigarillos are B, D, F, and H.

As shown in Fig 1 and Table 2, the mean values of PM concentration (C_mean_) for the reference cigarette and the cigarillos ranged from 2783 μg/m³ to 6686 μg/m³ for PM_10_, 2767 μg/m³ to 6585 μg/m³ for PM_2.5_, and 2441 to 4680 μg/m³ for PM_1_.

**Table 2:** Mean concentrations (C_mean_ PM_10_, PM_2.5_, and PM_1_) with standard deviation (SD) of all tested Tobacco products.

Brand	PM_10_ [μg/m³]	PM_2.5_ [μg/m³]	PM_1_ [μg/m³]
**Reference cigarette (f)**	4400 ± 520	4335 ± 504	3289 ± 891
**Cigarillo A**	6686 ± 650	6585 ± 628	4680 ± 405
**Cigarillo B (f)**	4124 ± 803	4103 ± 800	3429 ± 613
**Cigarillo C**	3989 ± 979	3933 ± 968	3069 ± 695
**Cigarillo D (f)**	2814 ± 588	2808 ± 585	2533 ± 452
**Cigarillo E**	5871 ± 1116	5760 ± 1034	4175 ± 419
**Cigarillo F (f)**	5549 ± 1467	5452 ± 1429	4001 ± 837
**Cigarillo G**	4213 ± 751	4187 ± 729	3486 ± 424
**Cigarillo H (f)**	2783 ± 392	2767 ± 384	2441 ± 304

The cigarillo brands A and B, C and D, E and F, and G and H are respectively pairs of the same brand without filter and with filter. f = filter-tipped tobacco product.

Compared to the measured PM_10_ values of the reference cigarette (C_mean_ = 4400 μg/m³), only cigarillo A showed significantly higher values (+ 52%, p = 0.0025). Cigarillo E (+ 33%, p = 0.1775) and cigarillo F (+ 26%, p > 0.9999) also showed higher values but without significance. The results were similar for PM_2.5_ (reference cigarette: 4335 μg/m³; A: +52%, p = 0.0017; E: +33%, p = 0.1623; F: +26%, p > 0.9999). For PM_1_ (reference cigarette: 3289 μg/m³), cigarillo A (+42%, p < 0.0001) and cigarillo E (+27%, p = 0.0487) showed higher values with significance, whereas cigarillo F (+22%, p > 0.9999) showed higher values but without significance. Regarding PM_10_, significantly lower measurement results were detected for cigarillo D (-36%, p = 0.0017) and cigarillo H (-37%, p = 0.0008) compared to the reference cigarette, regarding PM_2.5_, also for cigarillo D (-35%, p = 0.0021) and cigarillo H (-36%, p = 0.0009). In case of PM_1_, only cigarillo H showed significantly lower results (-26%, p = 0.0467), while cigarillo D showed 23% lower values than the reference cigarette but without significance (p = 0.1365). Similar measurement results compared to the reference cigarette were observed for cigarillo B (PM_10_: -6%, PM_2.5_: -5%, PM_1_: +4%), cigarillo C (PM_10_: -9%, PM_2.5_: -9%, PM_1_: -7%), and cigarillo G (PM_10_: -4%, PM_2.5_: -3%, PM_1_: +6%).

All filter-tipped cigarillos (B, D, F, H) showed lower PM measurement results than their counterparts of the same brand without filter, with cigarillos D and F showing no significance. The range at PM_10_ and PM_2.5_ were between 38% and 5% lower PM emissions and between 30% and 4% in the case of PM_1_, respectively. A detailed overview of the percentage differences and the probability values (p) is shown in Table 3.

**Table 3:** PM mean value comparison of the cigarillos with filter (B, D, F, H) and the counterparts of the same brand with no filter regarding percentage differences and probability values (p).

Cigarillo pair	Percentage difference	p-value
B vs. A (PM_10_)	-38%	< 0.0001
B vs. A (PM_2.5_)	-38%	0.0001
B vs. A (PM_1_)	-27%	0.0006
D vs. C (PM_10_)	-29%	0.1454
D vs. C (PM_2.5_)	-29%	0.1896
D vs. C (PM_1_)	-17%	> 0.9999
F vs. E (PM_10_)	-5%	> 0.9999
F vs. E (PM_2.5_)	-5%	> 0.9999
F vs. E (PM_1_)	-4%	> 0.9999
H vs. G (PM_10_)	-34%	0.0078
H vs. G (PM_2.5_)	-34%	0.0058
H vs. G (PM_1_)	-30%	0.0053

Significance level was set at p < 0.05. All statistical data based on Dunn’s multiple comparison test.

**Fig 2:**
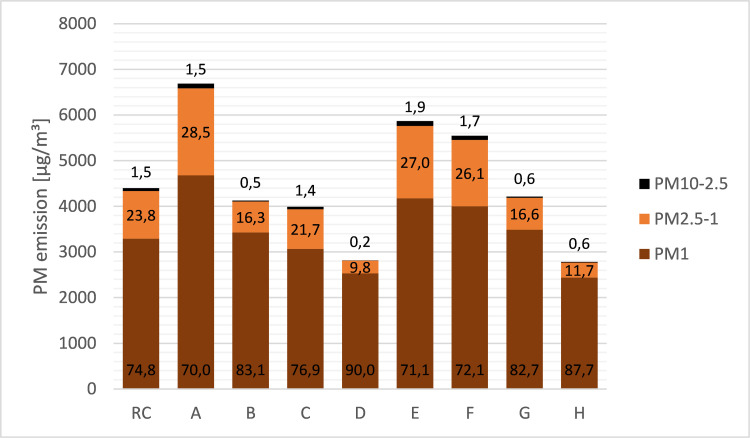
Distribution pattern of PM_10-2.5_, PM_2.5–1_, and PM_1_ in the second-hand smoke of all investigated tobacco products.

As seen in Fig 2, most particles detected belonged to the PM_1_ fraction (70% to 90%). Depending on the tobacco product, only 0.2% to 1.9% were coarse particles (fraction PM_10-2.5_).

## Discussion

Real cigarillos can cause very high PM levels. The average PM concentrations we observed were up to 1.5 times higher than the PM concentrations from the filter-tipped reference cigarette 3R4F. The measured values of PM_2.5_ and PM_10_ (+52%), in particular, of cigarillo A were higher than those of the reference cigarette. Also, the PM_1_ values (+42%) of cigarillo A were substantially higher. Of all eight cigarillo brands tested, three brands showed notable higher PM emissions than the reference cigarette. Two cigarillo brands emitted less PM, and three brands showed similar PM values compared to the reference cigarette. These variations of PM emissions relative to the reference cigarette are consistent with previous ToPIQ studies on cigarettes, where different brands showed likewise more or fewer PM emissions compared to the reference cigarette within a similar range of percentage variation [29,31,34]. Our results differ slightly from studies of particle emissions of six cigars, three cigarillos, and four cigarettes performed in an unventilated 20 m³ chamber [35]. The authors found the lowest total particle mass values and emission rates (mg/min respectively mg/g-smoked) in the cigarillos, albeit of the same brand. In contrast, in another study, Lawyer et al. showed substantially higher PM concentrations of all flavored cigarillos tested compared to the reference cigarettes 1R6F and especially 3R4F [36].

All cigarillos without filter showed higher PM values than the filtered counterparts of the same brand. Since a filter represents a drawing resistance during smoking, this could have led to a different burning and smoldering of the tobacco with lower PM emission, especially in the side-stream smoke. Lower oxygen feed might have resulted in poor combustion of the tobacco at lower temperatures leading to higher PM emissions. Probably, some PM, particularly coarse particles, were likely to be retained in the filter and not released as exhaled mainstream smoke. In contrast to PM concentrations in mainstream smoke, it was reported that filter types (e.g., ventilation grade) or puff volume have only a few effects on particle emissions in side-stream smoke [37]. This should be taken into account in future studies.

More detailed information, such as the amount of nicotine, tar, CO, or additives, is unfortunately not available for cigars and cigarillos from the BMEL database (Table 1) [27]. In the scientific literature, research on the ingredients of cigars and cigarillos is very limited. However, substantially higher amounts of nicotine, volatile, and semi-volatile compounds than in conventional cigarettes have been reported [38,39]. That is all the more incomprehensible since cigarillos are often flavored with various additives [36]. By the directive 2014/40/EC of the European Parliament and the European Council, the measurement and declaration of nicotine, tar, and CO in mainstream smoke are mandatory for cigarette brands, but not for cigars or cigarillos [40]. Due to a lack of information on the ingredients of the investigated cigarillo brands, the causes for the varying PM emissions cannot be distinctly identified. Reasons could be different tobacco strength, additives, or the texture of the tobacco leaf. In any case, the provision of more information for both cigars and cigarillos, especially the declaration of the amounts of nicotine, tar, and CO, is desirable and necessary. Since this study showed high emissions of the particularly hazardous fine particles smaller than 2.5 μm, the declaration of the PM emissions should be mandatory for all tobacco products. A consistent and comprehensive declaration of mainstream and side-stream smoke emissions by manufacturers and importers is useful for health authorities and policy-makers.

The WHO Air quality guidelines recommend that the 24-hour mean concentration of PM_2.5_ should not exceed 25 μg/m³ [41]. The PM_2.5_ measuring data of the investigated tobacco products show values up to more than 250-fold. Semple and Latif [42] analyzed PM_2.5_ from SHS in smokers’ homes. They found an average time of one hour for the PM values to be halved. Ergo, it would take hours until the PM load in the measuring cabin will fall below the 25 μg/m³ mark without ventilation. It should be noted that the cabin with an indoor volume of 2.88 m³ corresponds to the passenger and cargo volume of a compact car (2.832 to 3.087 m³) according to US EPA [43]. Therefore, the conditions in the measuring cabin and a compact car with closed windows and turned-off ventilation are comparable. PM burden by SHS would be very high and long-lasting. The harmfulness of PM in SHS must always be emphasized. It also affects car passengers, who are often children. Not least because for this reason, a global ban on smoking in cars is desirable.

The LAS Grimm model 1.109 detects via light scattering small particles from a diameter of 0.25 μm and, consequently, no particles between 0.1 μm and 0.25 μm. However, they also belong to the PM_1_ fraction. This technical limitation must be mentioned because a large proportion of the particles in tobacco smoke is smaller than 1 μm. Several studies described different particle sizes of tobacco smoke, e.g., 0.1–1 μm, 0.02–2 μm, or geometric mean diameters between 0.1 and 0.5 μm [35,44–47]. Therefore, the measured values of this study may be somewhat too low. Nevertheless, the LAS used is capable of detecting most of the PM emissions from SHS.

Since the sample air had to be diluted with compressed air, collecting samples on filters for gravimetric measurements was not possible because the diluting system (Grimm VKL mini 7.951) was connected to the LAS at the location of the filter holder. The reference methods for PM measuring of the US EPA (Federal Reference Methods, FRMs) and the European Committee for Standardization (EN 12341) are often gravimetric methods measuring the PM_10_ and PM_2.5_, but not the PM_1_ mass concentrations. Both are based on a 24 h sample collection on filters and subsequently weighing the collected PM [48,49]. The Tapered Element Oscillating Microbalance (TEOM) Monitor and the Grimm model EDM 180, which also measures via light scattering, are FRMs, too. The PM measurement results of the Grimm model 1.109 used are very similar to those of gravimetric methods, TEOM monitors, or the Grimm model EDM 180 [50]. According to a study by Fromme et al. [51], the measured PM values of LAS are generally lower than the results of gravimetric methods, but the rank order of the measured data correlates very strongly. Hence, the generated data of a Grimm model 1.109 can be considered valid. In addition, this device has the advantage of allowing PM measuring, including semi-volatile fractions, of each single tobacco product in real-time [50]. It is essential to maintain the measuring method during the investigation.

A limitation of this study is that the applied AETSE cannot exactly imitate human smoking behavior and thus SHS. On the one hand, this is due to the humidification of inhaled mainstream smoke in the human respiratory tract and the resulting 1.5-fold larger particle size of the exhaled smoke by hygroscopic growth [52]. Moreover, in the literature, a wide margin (average 40–97%) regarding the retention rate of particles in the human respiratory tract is reported [53]. Therefore, the presented PM amounts could be slightly too high with a shift to smaller particles compared to a set-up with a human smoker. However, since SHS caused by human smokers is composited of only about 15% mainstream smoke and 85% side-stream smoke [54,55], the smoke produced by the AETSE is very similar to SHS.

The WHO-formulated Standard Operating Procedure for Intense Smoking of Cigarettes (puff volume 55 ml; two puffs/min; completely blocked filter ventilation holes) [56] or the ISO Standard for the Machine Smoking of Cigarettes by the International Organization of Standardization (puff volume 35 ml; one puff/min; non-blocked filter ventilation holes) [57] are existing protocols to name two smoking regimes. However, there is no “gold standard” for smoking regimes [58]. The experimental design used here differs from these protocols. The smoking of tobacco products was conducted in a measuring cabin rather than with a smoking machine with the advantage of a test volume that more closely resembles the reality of smoking. However, this resulted in a limited ability to calibrate the set-up. The applied modified protocol (8 regular puffs; puff volume 40 ml; two puffs/min; non-blocked filter ventilation holes) represents a compromise of existing protocols [59,60] and was also developed by observations of real smokers [61]. However, the regime used provides reliable and reproducible results without the risk of tobacco smoke exposition to the investigator or a test person.

The main focus in this study, as in previous ToPIQ-studies, was on the comparison of PM data between the investigated tobacco products and the reference cigarette [13,14,29,31,34,61]. Reporting the percentage deviation to the reference cigarette allows comparing the results of ToPIQ-studies with each other. Therefore, the modified method applied can be considered valid. Absolute values are reported to compare PM data with other studies or guidelines. Thereby, the level of PM burden caused by SHS can be shown in a demonstrative way.

## Conclusions

Our data show the massive PM burden caused by smoking, especially in interiors. Unfiltered cigarillos may release more PM than regular cigarettes or filter-tipped cigarillos. The reasons for the emission differences could not be clarified distinctly due to a lack of information on the part of the cigarillo manufacturers. To protect smokers and non-smokers, it should be mandatory for tobacco companies and importers of tobacco products to declare the PM emissions in mainstream and side-stream smoke of all tobacco products to government officials and public authorities. A consistent and comprehensive declaration of emissions should be obligatory. Further studies on PM emissions from all tobacco products, including cigarillos and cigars, are reasonable and necessary.

## Abbreviations

AETSE: Automatic Environmental Tobacco Smoke Emitter
ANOVA: Analysis of variance
C_mean_: Mean concentration
CO: Carbon monoxide
EN: European norm
EPA: Environmental Protection Agency
FRM: Federal Reference Method
LAS: Laser Aerosol Spectrometer
NCD: Non-communicable disease
PM: Particulate matter
SHS: Second-hand smoke
TEOM: Tapered Element Oscillating Microbalance
ToPIQ: Tobacco Smoke Particles and Indoor Air Quality
WHO: World Health Organization
